# Pythagorean 2-Tuple Linguistic Taxonomy Method for Supplier Selection in Medical Instrument Industries

**DOI:** 10.3390/ijerph16234875

**Published:** 2019-12-03

**Authors:** Tingting He, Guiwu Wei, Jianping Lu, Cun Wei, Rui Lin

**Affiliations:** 1 School of Business, Sichuan Normal University, Chengdu 610101, China; m_hetingting@163.com (T.H.); lujp2002@163.com (J.L.); 2 School of Statistics, Southwestern University of Finance and Economics, Chengdu 611130, China; weicun1990@163.com; 3 School of Economics and Management, Chongqing University of Arts and Sciences, Chongqing 402160, China

**Keywords:** multiple attribute group decision making (MAGDM), Pythagorean 2-tuple linguistic numbers (P2TLNs), Taxonomy method, supplier selection, medical instrument industries

## Abstract

Supplier selection in medical instrument industries is a classical multiple attribute group decision making (MAGDM) problem. The Pythagorean 2-tuple linguistic sets (P2TLSs) can reflect uncertain or fuzzy information well and solve the supplier selection in medical instrument industries, and the original Taxonomy is very appropriate for comparing different alternatives with respect to their advantages from studied attributes. In this study, we present an algorithm that combines Pythagorean 2-tuple linguistic numbers (P2TLNs) with the Taxonomy method, where P2TLNs are applied to express the evaluation of decision makers on alternatives. Relying on the Pythagorean 2-tuple linguistic weighted average (P2TLWA) operator or Pythagorean 2-tuple linguistic weighted geometric (P2TLWG) operator to fuse P2TLNs, the new general framework is established for Pythagorean 2-tuple linguistic multiple attribute group decision making (MAGDM) under the classical Taxonomy method. Ultimately, an application case for supplier selection in medical instrument industries is designed to test the novel method’s applicability and practicality and a comparative analysis with three other methods is used to elaborate further.

## 1. Introduction

The fuzzy set theory [1] was first introduced to describe the uncertainty and fuzziness of things. In order to reflect the objective world as faithfully as possible, many people offered some extended forms of the fuzzy set, such as interval-valued hesitant fuzzy sets (IVHFSs), type-2 fuzzy sets (T2FSs), and intuitionistic fuzzy sets (IFSs) [2]. The IFS theory was proposed by Atanassov [2] in 1986 as an important extension of the classical fuzzy set theory. The research on its theory and application has achieved extensive research results in the field of fuzzy set theory [3,4,5,6,7,8,9]. However, when using IFSs to make decisions, the following situation may occur: the membership degree plus the non-membership degree of the scheme satisfying attributes given by the decision makers is greater than 1. Based on this, in 2013, American scholar Yager [10] proposed the Pythagorean fuzzy set (PFSs), which makes membership degree plus non-membership degree greater than 1, but the sum of squares does not exceed 1. Therefore, the decision maker (DM) does not need to modify the values of membership and non-membership, can be more accurate and gives a detailed description of the reality [11,12,13,14,15,16,17].

After the PFSs were proposed, a large number of researchers combined the PFSs [10] with various methods and applied these proposed methods to multiple attribute decision making (MADM) issues. Zhang and Xu [18] firstly put forward the mathematical expression of PFSs, and then they tied the PFSs and Technique for Order of Preference by Similarity to Ideal Solution (TOPSIS) method together. Zhang [19] presented a Pythagorean fuzzy QUALItative FLEXible multiple criteria method (QUALIFLEX) method with the closeness index to address the layered multi-criteria decision-making issue under PFSs on the basis of PFNs and interval-valued Pythagorean fuzzy numbers (IVPFNs). Ren et al. [20] provided a case of choosing the governor of Asian Infrastructure Investment Bank by using the Pythagorean fuzzy TODIM (PF-TODIM) method to observe the feasibility of the model. Bolturk [21] expanded the COmbinative Distance-based Assessment (CODAS) model to PFSs to propose a novel method, which is PF-CODAS. They addressed a MADM problem of supplier selection utilizing the new method to show its validity and effectiveness. Chen [22] defined a new VIseKriterijumska Optimizacija I KOmpromisno Resenje (VIKOR)-based method for MADM analysis containing PFSs. PFSs have certain advantages over un-normalized fuzzy sets such as IFS in dealing with fuzziness and complex uncertainty. Based on this, a Pythagorean fuzzy VIKOR method based on distance index is proposed, which is quite different from the existing VIKOR method. Huang and Wei [23] put forward a new extended TODIM to deal with the MADM issue. Ilbahar et al. [24] proposed the three methods of Fine Kinney, Pythagorean fuzzy analytic hierarchy process and PFPRA. Khan et al. [25] presented an extension of TOPSIS under the interval value Pythagorean fuzzy context, using the interval-valued Pythagorean fuzzy Choquet integral geometric (IVPFCIG) operator and distance formula based on the Choquet integral to aggregate all fuzzy decision matrixes. Perez-Dominguez et al. [26] combined ratio analysis-based multiple objective optimization under PFSs to select an appropriate alternative. A novel Linear Programming Technique for Multidimensional Analysis of Preference (LINMAP) method was expanded by Xue et al. [27] to the PFSs.

The Taxonomy method was originally proposed by Adanson in 1763 and was developed in 1950 by a group of Polish mathematicians. In 1968, Zyegnant Hellwing from the Wroclaw High School released this method as a method of classifying and determining the degree of development. Based on the state’s level of development and resources, and the structure of skilled employees, Hellwig [28] applied the method to national classifications. Afterwards, Hellwig [29] evaluated high-level manpower by means of this method. Due to the internal diversity of Germany in terms of social and economic development, Barbara Jurkowska [30] utilized the Taxonomy method to analyze the level of German social and economic development, making it possible to determine the development direction of a particular country. Bienkowska [31] did a study that used the Taxonomy method to determine the level of development of Polish local municipalities and the actions the authorities took to promote entrepreneurship.

Based on previous studies, this paper tries to propose a new approach, so we joined the 2-tuple linguistic variable to Pythagorean fuzzy numbers and then combined it with the Taxonomy method. This paper utilized the P2TL-Taxonomy model for supplier selection in medical instrument industries by using relevant assessment criteria. Our goal in this article is to combine the origin Taxonomy with Pythagorean 2-tuple linguistic numbers (P2TLNs) to address MAGDM issues. The innovativeness of the paper can be summarized as follows: (1) the Taxonomy method is extended by P2TLSs; (2) the Pythagorean 2-tuple linguistic Taxonomy (P2TL-Taxonomy) method is proposed to solve the Pythagorean 2-tuple linguistic MAGDM problems; (3) a numerical example for supplier selection in medical instrument industries is supplied to show the developed approach; and (4) some comparative studies are provided with the existing methods to give effect to the rationality of P2TL-Taxonomy.

The remainder of this article is organized as follows: Section 2 introduces some basic definitions of P2TLNs; Section 3 extends the Taxonomy method with P2TLNs; in Section 4, a case study for supplier selection in medical instrument industries and contrastive analysis is given; and Section 5 supplies the conclusions.

## 2. Preliminaries

### 2.1. Pythagorean 2-Tuple Linguistic Sets

Wei et al. [23] proposed the Pythagorean 2-tuple linguistic sets (P2TLSs) based on the PFSs [32] and 2-tuple linguistic information [33].

**Definition** **1** *[23]. A P2TLS O in X is given*

(1) $$O=\left\{\left(s_{\sigma (x)},\psi \right),\left(u_{o}\left(x\right),\nu_{o}\left(x\right)\right),x\in X\right\}$$

*where $s_{\sigma (x)}\in S$, ψ∈[−0.5，0.5),$u_{o}\left(x\right)\in [0,1]$ and $v_{o}\left(x\right)\in [0,1]$, $u_{o}\left(x\right)$ and $\nu_{o}\left(x\right)$ satisfy the following condition $0\leq {\left(u_{o}\left(x\right)\right)}^{2}+{\left(v_{o}\left(x\right)\right)}^{2}\leq 1$, ∀x∈X. The numbers $u_{o}\left(x\right),\nu_{o}\left(x\right)$ represent the degree of membership and degree of non-membership of the element x to linguistic variable $\left(s_{\sigma (x)},\psi \right)$.*

$O=\langle \left(s_{\sigma },\psi \right),\left(u_{o},v_{o}\right)\rangle$ is called a Pythagorean 2-tuple linguistic number (P2TLN).

**Definition** **2** *[23]. Suppose that $o=\langle \left(s_{\sigma },\psi \right),\left(u_{o},v_{o}\right)\rangle$ is a P2TLN, the score function of P2TLN can be depicted as follows:*

(2) $$\begin{array}{l} S(o)=\Delta \left(\Delta^{-1}\left(s_{\sigma \left(o\right)},\psi \right)\frac{1+{\left(u_{o}\right)}^{2}-{\left(\nu_{o}\right)}^{2}}{2}\right),\\ \Delta^{-1}\left(S\left(o\right)\right)\in \left[0,L\right]. \end{array}$$

**Definition** **3** *[23]. Suppose that $o=\langle \left(s_{\sigma },\psi \right),\left(u_{o},v_{o}\right)\rangle$ is a P2TLN, the accuracy function of P2TLN can be depicted as follows:*

(3) $$\begin{array}{l} H(o)=\Delta \left(\Delta^{-1}\left(s_{\varphi \left(o\right)},\rho \right)\frac{{\left(u_{o}\right)}^{2}+{\left(\nu_{o}\right)}^{2}}{2}\right),\\ \Delta^{-1}\left(H\left(o\right)\right)\in \left[0,L\right]. \end{array}$$

**Definition** **4** *[23]. Suppose that $o_{1}=\langle \left(s_{\sigma_{1}},\psi_{1}\right),\left(u_{o_{1}},v_{o_{1}}\right)\rangle$ and $o_{2}=\langle \left(s_{\sigma_{2}},\psi_{2}\right),\left(u_{o_{2}},v_{o_{2}}\right)\rangle$ are two P2TLNs. Respectively, the scores of $o_{1}$ and $o_{2}$ are $S\left(o_{1}\right)=\Delta \left(\Delta^{-1}\left(s_{\sigma (o_{1})},\psi_{1}\right)\cdot \frac{1+{\left(u_{o_{1}}\right)}^{2}-{\left(\nu_{o_{1}}\right)}^{2}}{2}\right)$ and $S\left(o_{2}\right)=\Delta \left(\Delta^{-1}\left(s_{\sigma (o_{2})},\psi_{2}\right)\cdot \frac{1+{\left(u_{o_{2}}\right)}^{2}-{\left(\nu_{o_{2}}\right)}^{2}}{2}\right)$, and let $H\left(o_{1}\right)=\Delta \left(\Delta^{-1}\left(s_{\sigma (o_{1})},\psi_{1}\right)\cdot \frac{{\left(u_{o_{1}}\right)}^{2}+{\left(\nu_{o_{1}}\right)}^{2}}{2}\right)$ and $H\left(o_{2}\right)=\Delta \left(\Delta^{-1}\left(s_{\sigma (o_{2})},\psi_{2}\right)\cdot \frac{{\left(u_{o_{2}}\right)}^{2}+{\left(\nu_{o_{2}}\right)}^{2}}{2}\right)$ be the accuracy degrees of $o_{1}$ and $o_{2}$, then some operational laws of P2TLNs can be defined as follows:*

(1) $if\;S\left(o_{1}\right)<S\left(o_{2}\right),o_{1}<o_{2};$
(2) $if\;S\left(o_{1}\right)>S\left(o_{2}\right),o_{1}>o_{2};$
(3) $if\;S\left(o_{1}\right)=S\left(o_{2}\right),H\left(o_{1}\right)<H\left(o_{2}\right),\;then\;o_{1}<o_{2};$
(4) $if\;S\left(o_{1}\right)=S\left(o_{2}\right),H\left(o_{1}\right)>H\left(o_{2}\right),\;then\;o_{1}>o_{2};$
(5) $if\;S\left(o_{1}\right)=S\left(o_{2}\right),H\left(o_{1}\right)=H\left(o_{2}\right),\;then\;o_{1}=o_{2};$

**Definition** **5** *[23]. Suppose that $o_{1}=\langle \left(s_{\sigma_{1}},\psi_{1}\right),\left(u_{o_{1}},v_{o_{1}}\right)\rangle$ and $o_{2}=\langle \left(s_{\sigma_{2}},\psi_{2}\right),\left(u_{o_{2}},v_{o_{2}}\right)\rangle$ are two P2TLNs, the normalized Hamming distance ($H_{d}$) between $a_{1}$ and $a_{2}$ can be depicted below:*

(4) $$H_{d}\left(o_{1},o_{2}\right)=\frac{1}{2L}\left[\left|\begin{array}{l} \left(1+{\left(u_{o_{1}}\right)}^{2}-{\left(v_{o_{1}}\right)}^{2}\right)\cdot \Delta^{-1}\left(s_{\sigma_{1}},\psi_{1}\right)-\\ \left(1+{\left(u_{o_{2}}\right)}^{2}-{\left(v_{o_{2}}\right)}^{2}\right)\cdot \Delta^{-1}\left(s_{\sigma_{2}},\psi_{2}\right) \end{array}\right|\right]$$

*where L represents the length of the language scale. It is a numerical value.*

**Definition** **6** *[23]. Suppose that $o_{1}=\langle \left(s_{\sigma_{1}},\psi_{1}\right),\left(u_{o_{1}},v_{o_{1}}\right)\rangle$ and $o_{2}=\langle \left(s_{\sigma_{2}},\psi_{2}\right),\left(u_{o_{2}},v_{o_{2}}\right)\rangle$ are two P2TLNs, then*

*$\begin{array}{l} o_{1}⊕o_{2}=\langle \Delta \left(\Delta^{-1}\left(s_{\sigma_{1}},\psi_{1}\right)+\Delta^{-1}\left(s_{\sigma_{2}},\psi_{2}\right)\right),\left(\sqrt{{\left(u_{o_{1}}\right)}^{2}+{\left(u_{o_{2}}\right)}^{2}-{\left(u_{o_{1}}\right)}^{2}{\left(u_{o_{2}}\right)}^{2}},\nu_{o_{1}}\nu_{o_{2}}\right)\rangle ;\\ o_{1}⊗o_{2}=\langle \Delta \left(\Delta^{-1}\left(s_{\sigma_{1}},\psi_{1}\right)\cdot \Delta^{-1}\left(s_{\sigma_{2}},\psi_{2}\right)\right),\left(u_{o_{1}}u_{o_{2}},\sqrt{{\left(\nu_{o_{1}}\right)}^{2}+{\left(\nu_{o_{2}}\right)}^{2}-{\left(\nu_{o_{1}}\right)}^{2}{\left(\nu_{o_{2}}\right)}^{2}}\right)\rangle ;\\ \lambda o_{1}=\langle \Delta \left(\lambda \Delta^{-1}\left(s_{\sigma_{1}},\psi_{1}\right)\right),\left(\sqrt{1-{\left(1-{\left(u_{o_{1}}\right)}^{2}\right)}^{\lambda }},{\left(\nu_{o_{1}}\right)}^{\lambda }\right)\rangle ;\\ {\left(o_{1}\right)}^{\lambda }=\langle \Delta \left({\left(\Delta^{-1}\left(s_{\sigma_{1}},\psi_{1}\right)\right)}^{\lambda }\right),\left({\left(u_{o_{1}}\right)}^{\lambda }，\sqrt{1-{\left(1-{\left(\nu_{o_{1}}\right)}^{2}\right)}^{\lambda }}\right)\rangle . \end{array}$*

**Theorem** **1** *[23]. For any two P2TLNs $o_{1}=\langle \left(s_{\sigma_{1}},\psi_{1}\right),\left(u_{o_{1}},v_{o_{1}}\right)\rangle$ and $o_{2}=\langle \left(s_{\sigma_{2}},\psi_{2}\right),\left(u_{o_{2}},v_{o_{2}}\right)\rangle$, according to Definition 6, we can naturally get the following properties of the operation laws:*

(1) $o_{1}⊕o_{2}=o_{2}⊕o_{1}$
(2) $o_{1}⊗o_{2}=o_{2}⊗o_{1}$
(3) $k\left(o_{1}⊕o_{2}\right)=ko_{1}⊕ko_{2},0\leq k\leq 1$
(4) $k_{1}o_{1}⊕k_{2}o_{1}=\left(k_{1}⊕k_{2}\right)o_{1},0\leq k_{1},k_{2},k_{1}+k_{2}\leq 1$
(5) $o_{1}{}^{k_{1}}⊗o_{1}{}^{k_{2}}={\left(o_{1}\right)}^{k_{1}+k_{2}},0\leq k_{1},k_{2},k_{1}+k_{2}\leq 1$
(6) $o_{1}{}^{k_{1}}⊗o_{2}{}^{k_{1}}={\left(o_{1}⊗o_{2}\right)}^{k_{1}},k_{1}\geq 0$
(7) ${\left({\left(o_{1}\right)}^{k_{1}}\right)}^{k_{2}}={\left(o_{1}\right)}^{k_{1}k_{2}}$

### 2.2. Some Operators with P2TLNs

In this section, some operators with P2TLNs will be introduced, such as the Pythagorean 2-tuple linguistic weighted average (P2TLWA) operator and the Pythagorean 2-tuple linguistic weighted geometric (P2TLWG) operator.

**Definition** **7** *[23]. Assume that $o_{j}=\langle \left(s_{\sigma_{j}},\psi_{j}\right),\left(u_{o_{j}},v_{o_{j}}\right)\rangle \left(j=1,2,\ldots ,n\right)$ is a collection of P2TLNs, the P2TLWA operator can be depicted as follows:*

(5) $$\begin{array}{l} {P2\mathrm{TLWA}}_{\omega }\left(o_{1},o_{2},\ldots ,o_{n}\right)=\overset{n}{\underset{j=1}{⊕}}\left(\omega_{j}o_{j}\right)\\ =\langle \Delta \left(\sum_{j=1}^{n}\omega_{j}\Delta^{-1}\left(s_{\sigma_{j}},\psi_{j}\right)\right),\left(\sqrt{1-\prod_{j=1}^{n}{\left(1-{\left(u_{o_{j}}\right)}^{2}\right)}^{\omega_{j}}},\prod_{j=1}^{n}{\left(\nu_{o_{j}}\right)}^{\omega_{j}}\right)\rangle \end{array}$$

*where $\omega ={\left(\omega_{1},\omega_{2},\ldots ,\omega_{n}\right)}^{T}$ is the weight vector of $o_{j}\left(j=1,2,\ldots ,n\right)$ and $\omega_{j}>0,\sum_{j=1}^{n}\omega_{j}=1.$*

**Definition** **8** *[23]. Assume that $o_{j}=\langle \left(s_{\sigma_{j}},\psi_{j}\right),\left(u_{o_{j}},v_{o_{j}}\right)\rangle \left(j=1,2,\ldots ,n\right)$ is a collection of P2TLNs, the P2TLWG operator can be depicted as follows:*

(6) $$\begin{array}{l} {P2\mathrm{TLWG}}_{\omega }\left(o_{1},o_{2},\ldots ,o_{n}\right)=\overset{n}{\underset{j=1}{⊗}}\left(\omega_{j}o_{j}\right)\\ =\langle \Delta \left(\prod_{j=1}^{n}\Delta^{-1}{\left(s_{\sigma_{j}},\psi_{j}\right)}^{\omega_{j}}\right),\left(\prod_{j=1}^{n}{\left(u_{o_{j}}\right)}^{\omega_{j}}\sqrt{1-\prod_{j=1}^{n}{\left(1-{\left(\nu_{o_{j}}\right)}^{2}\right)}^{\omega_{j}}}\right)\rangle \end{array}$$

*where $\omega ={\left(\omega_{1},\omega_{2},\ldots ,\omega_{n}\right)}^{T}$ is the weight vector of $o_{j}\left(j=1,2,\ldots ,n\right)$ and $\omega_{j}>0,\sum_{j=1}^{n}\omega_{j}=1.$*

## 3. Taxonomy Method for P2TL-MAGDM Issues

Suppose that ${Ν}_{i}=\left\{{Ν}_{1},{Ν}_{2},\ldots {Ν}_{m}\right\}$ and $\kappa_{j}=\left\{\kappa_{1},\kappa_{2},\ldots \kappa_{n}\right\}$ are respectively m alternatives and n criteria. Let $\gamma_{j}$ be the criteria’s weighting vector that satisfies $\vartheta_{j}\in \left[0,1\right]$ and $\sum_{j=1}^{n}\vartheta_{j}=1$. Let $\Theta =\left\{\Theta_{1},\Theta_{2},\ldots \Theta_{k}\right\}$ be the group of DMs, $\gamma =\left\{\gamma_{1},\gamma_{2},\ldots \gamma_{k}\right\}$ be the weight of DMs, with $\gamma_{t}\in \left[0,1\right]$ and $\sum_{t=1}^{k}\gamma_{t}=1$. Construct a decision matrix $X^{\left(t\right)}={\left(x_{ij}^{\left(t\right)}\right)}_{m\times n}$, where $X^{\left(t\right)}={\left(x_{ij}^{\left(t\right)}\right)}_{m\times n}={\langle \left(s_{{}_{\sigma_{ij}}}^{\left(t\right)},\psi_{ij}^{\left(t\right)}\right),\left(u_{r_{ij}}^{\left(t\right)},v_{r_{ij}}^{\left(t\right)}\right)\rangle }_{m\times n}$ means the performance of the alternative ${Ν}_{i}\left\{i=1,2\cdots ,m\right\}$ with respect to criteria $\kappa_{j}\left\{j=1,2\cdots ,n\right\}$ by expert $\Theta^{\left(t\right)}\left\{t=1,2,\ldots k\right\}$ using a P2TLN, $0\leq u_{r_{ij}}^{\left(t\right)}\leq 1,\;$ $0\leq v_{r_{ij}}^{\left(t\right)}\leq 1$ and $0\leq {\left(u_{r_{ij}}^{\left(t\right)}\right)}^{2}+{\left(v_{r_{ij}}^{\left(t\right)}\right)}^{2}\leq 1，$ i=1,2⋯,m, j=1,2⋯,n, t=1,2⋯,k.

In view of both the P2TLN theories and procedures from the Taxonomy method [34], we put forward a P2TL-Taxonomy method to deal with the problem of MAGDM effectively. The new model can be shown below:

**Step 1.** Shift the cost attribute into the beneficial attribute.

**Step 2.** Set up a decision-making group composed of several experts, choose the best attributes to measure alternatives, and finally get a P2TL fuzzy decision matrix series $X^{\left(t\right)}={\left(x_{ij}^{\left(t\right)}\right)}_{m\times n}$ from each decision maker.

(7) $$X^{\left(t\right)}={\left[x_{ij}^{\left(t\right)}\right]}_{m\times n}={\left[\begin{array}{ccccc} x_{11}^{\left(t\right)} & \cdots & x_{1j}^{\left(t\right)} & \cdots & x_{1n}^{\left(t\right)}\\ \vdots & \ddots & \vdots & \ddots & \vdots \\ x_{21}^{\left(t\right)} & \cdots & x_{ij}^{\left(t\right)} & \cdots & x_{in}^{\left(t\right)}\\ \vdots & \ddots & \vdots & \ddots & \vdots \\ x_{m1}^{\left(t\right)} & \cdots & x_{mj}^{\left(t\right)} & \cdots & x_{mn}^{\left(t\right)} \end{array}\right]}_{m\times n}$$

where $x_{ij}^{\left(t\right)}$ denotes the fuzzy performance value of the i-th alternative (i=1,2,…,m) with respect to the j-th criterion (j=1,2,…,n) and the t-th decision-maker (t=1,2,…,k).

**Step 3.** Utilize the P2TLWA operator or the P2TLWG operator to fuse assessment information, then the P2TL fuzzy decision matrix $X={\left(x_{ij}\right)}_{m\times n}$ group can be obtained by the calculation.

(8) $$X_{ij}={\left[x_{ij}\right]}_{m\times n}={\left[\begin{array}{ccccc} x_{11}^{} & \cdots & x_{1j}^{} & \cdots & x_{1n}^{}\\ \vdots & \ddots & \vdots & \ddots & \vdots \\ x_{21}^{} & \cdots & x_{ij}^{} & \cdots & x_{in}^{}\\ \vdots & \ddots & \vdots & \ddots & \vdots \\ x_{m1}^{} & \cdots & x_{mj} & \cdots & x_{mn}^{} \end{array}\right]}_{m\times n}$$

(9) $$\begin{array}{l} x_{ij}=\overset{k}{\underset{t=1}{⊕}}x_{ij}^{\left(k\right)}=P2\mathrm{TLWA}(x_{ij}^{\left(1\right)},x_{ij}^{\left(2\right)},\ldots ,x_{ij}^{\left(k\right)})\\ =\langle \Delta \left(\sum_{t=1}^{k}\gamma_{t}\Delta^{-1}\left(s_{{}_{\sigma_{ij}}}^{\left(t\right)},\psi_{ij}^{\left(t\right)}\right)\right),\left(\sqrt{1-\prod_{t=1}^{k}{\left(1-{\left(u_{r_{ij}}^{\left(t\right)}\right)}^{2}\right)}^{\gamma_{t}}},\prod_{t=1}^{k}{\left(\nu_{r_{ij}}^{\left(t\right)}\right)}^{\gamma_{t}}\right)\rangle \end{array}$$

or

(10) $$\begin{array}{l} x_{ij}=\overset{k}{\underset{t=1}{⊗}}x_{ij}^{\left(k\right)}=P2\mathrm{TLWG}(x_{ij}^{\left(1\right)},x_{ij}^{\left(2\right)},\ldots ,x_{ij}^{\left(k\right)})\\ =\langle \Delta \left(\prod_{t=1}^{k}\Delta^{-1}{\left(s_{{}_{\sigma_{ij}}}^{\left(t\right)},\psi_{ij}^{\left(t\right)}\right)}^{\gamma_{t}}\right),\left(\prod_{t=1}^{k}{\left(u_{r_{ij}}^{\left(t\right)}\right)}^{\gamma_{t}},\sqrt{1-\prod_{t=1}^{k}{\left(1-{\left(\nu_{r_{ij}}^{\left(t\right)}\right)}^{2}\right)}^{\gamma_{t}}}\right)\rangle \end{array}$$

where $x_{ij}$ means the average fuzzy performance value of the i-th alternative relative to the j-th criterion.

**Step 4.** Equations (11) and (12) are used to calculate the mean and standard deviation of the attributes.

(11) $${\bar{x}}_{j}=\frac{1}{m}\sum_{i=1}^{m}x_{ij}=\left\{\Delta \left(\sum_{j=1}^{n}\frac{1}{m}\Delta^{-1}\left(s_{\sigma_{j}},\psi_{j}\right)\right),\left(\sqrt{1-\prod_{j=1}^{n}{\left(1-{\left(u_{x_{j}}\right)}^{2}\right)}^{\frac{1}{m}}},\prod_{j=1}^{n}{\left(\nu_{x_{j}}\right)}^{\frac{1}{m}}\right)\right\}$$

(12) $$S_{j}=\sqrt{\frac{1}{m}\sum_{i=1}^{m}{\left(x_{ij}-{\bar{x}}_{j}\right)}^{2}}=\sqrt{\frac{1}{m}\sum_{i=1}^{m}H_{d}{\left(x_{ij},{\bar{x}}_{j}\right)}^{2}}$$

In Equation (12), $H_{d}$ means the normalized Hamming distance between two P2TLNs.

**Step 5.** The standard matrix:

(13) $$A_{ij}=\frac{x_{ij}-{\bar{x}}_{j}}{S_{j}}=\frac{H_{d}\left(x_{ij},{\bar{x}}_{j}\right)}{S_{j}}$$

(14) $$A_{ij}={\left[a_{ij}\right]}_{m\times n}={\left[\begin{array}{ccccc} a_{11}^{} & \cdots & a_{1j}^{} & \cdots & a_{1n}^{}\\ \vdots & \ddots & \vdots & \ddots & \vdots \\ a_{21}^{} & \cdots & a_{ij}^{} & \cdots & a_{in}^{}\\ \vdots & \ddots & \vdots & \ddots & \vdots \\ a_{m1}^{} & \cdots & a_{mj} & \cdots & a_{mn}^{} \end{array}\right]}_{m\times n};i=1,\ldots ,m,\;j=1,\ldots ,n$$

**Remark.**  *In the decision matrix, the alternatives are represented based on attributes with different metrics, such as the unit differences. Therefore, this step uses Equation (13) to change the decision matrix $X_{ij}$ to a standard matrix $A_{ij}$. Here, $a_{ij}$ denotes the standardized performance value of the i-th alternative in the j-th attributes.*

**Step 6:** The composite distances matrix:

(15) $$C_{ab}=\sqrt{\sum_{j=1}^{n}\vartheta_{j}{\left(z_{aj}-z_{bj}\right)}^{2}}$$

(16) $$C_{ab}={\left[c_{ab}\right]}_{m\times m}={\left[\begin{array}{ccccc} c_{11} & \cdots & c_{1b} & \cdots & c_{1m}\\ \vdots & \ddots & \vdots & \ddots & \vdots \\ c_{a1} & \cdots & c_{ab} & \cdots & c_{am}\\ \vdots & \ddots & \vdots & \ddots & \vdots \\ c_{m1} & \cdots & c_{mb} & \cdots & c_{mm} \end{array}\right]}_{m\times m}$$

**Remark.**  *In this step, use Equation (15) to calculate the distance between each alternative and the other alternative under each attribute. We will get a composite distance matrix $C_{ab}$ between the alternatives.*

**Step 7:** Homogenizing the alternatives:

(17) $$\bar{Q}=\frac{1}{m}\sum_{i=1}^{m}Q_{i}$$

(18) $$S_{Q}=\sqrt{\frac{1}{m}\sum_{i=1}^{m}{\left(Q_{i}-\bar{Q}\right)}^{2}}$$

(19) $$Q=\bar{Q}\pm 2S_{Q}$$

**Remark.**  *The minimum distance of each row is selected by the composite distance matrix determined in the previous step. Then, calculate the mean and standard deviation of the minimum distance value for each line according to Equation (17) and Equation (18). The homogeneity range of the composite distance matrix is calculated based on the mean and standard deviation according to Equation (19). If the minimum distance value for each row is not within the range, then they are inhomogeneous and eliminated, and then the mean and standard deviation of the values are calculated again.*

**Step 8:** The development pattern:

(20) $$C_{io}=\sqrt{\sum_{j=1}^{n}\vartheta_{j}{\left(z_{ij}-z_{oj}\right)}^{2}}$$

**Remark.**  *In this step, through Equation (19), using the matrix $A_{ij}$ obtained in **Step 4**, the alternative development pattern is determined, where $z_{oj}$ represents the ideal value for the j-th attribute. If $\kappa_{j}$ is a cost attribute, $z_{oj}$ is the minimum value of this column. Conversely, if $\kappa_{j}$ is a benefit attribute, $z_{oj}$ selects the maximum value of this column. $z_{ij}$ indicates the standardized value of the j-th attribute for the i-th alternative, and $C_{io}$ illustrates the development pattern for the i-th attribute.*

**Step 9:** The final ranking of alternatives:

(21) $$C_{O}={\bar{C}}_{io}+2S_{C_{io}};i=1,\cdots ,m$$

(22) $$F_{i}=\frac{C_{io}}{C_{O}};\;i=1,\cdots ,m$$

**Remark.**  *According to the mean and standard deviation of $C_{io}$, the high limit of development $C_{O}$ is initially calculated. The number of $F_{i}$ obtained from Equation (22) is between 0 and 1, and the smaller the value, the higher the ranking.*

## 4. Numerical Example and Comparative Analysis

### 4.1. Numerical Example

Supply chain management can effectively integrate the internal and external resources of an enterprise. Through planning, coordinating and controlling the logistics information flow and capital flow among suppliers, manufacturers, distributors and retailers in the whole supply chain, it can achieve the purpose of seeking more reasonable and efficient utilization of resources to maximize profits. The supplier as a business partnership is gaining more and more attention by the enterprise, so supplier evaluation and selection has been an important problem in supply chain management in the past. In regards to supplier evaluation and selection methods, whether it is a subjective judgment method, cost method, analytic hierarchy process, etc., most are aimed at the effect of the quality of the product or service provided by the supplier, but they seldom pay attention to the process of suppliers to provide products and services. In modern quality management, enterprises must pay attention to the process of suppliers to ensure product quality and pursue excellence. At the international level, GMP (good manufacturing practice) has become the basic principle of the drug manufacturing process and quality management. However, in the medical device industry, existing customers evaluate suppliers according to ISO13485 quality management system standards. Only by continuously evaluating the process of controlling the supplier can the results of the supplier’s product and service delivery be effectively guaranteed. How to introduce the process control method of the pharmaceutical industry into the medical device industry to strengthen the process control of products and reduce the risk of injury to patients caused by poor medical devices is very important. We hope that the commonly used food and drug GMP management process validation is introduced into the medical equipment supplier evaluation and selection. At the same time, we would like to see the supplier evaluation and selection of the commonly used Taxonomy method combined with other aspects of the quality factors of comprehensive evaluation and selection to suppliers. On the one hand, we hope to improve the process control ability of China’s medical equipment supplier, and on the other hand, we hope to introduce the theory of process validation for the medical devices industry. Supplier selection in medical instrument industries is a classical MAGDM issue [35,36,37,38]. In this section, we will design a numerical case for supplier selection in medical instrument industries by using the P2TL-Taxonomy model. Assume that five possible suppliers of medical instrument industries $N_{i}\left(i=1,2,3,4,5\right)$ and four evaluation criteria $\kappa_{j}\left(j=1,2,3,4\right)$ to evaluate these suppliers of medical instrument industries are selected: ① $\kappa_{1}$ is the environmental improvement quality; ② $\kappa_{2}$ is the transportation cost of suppliers; ③ $\kappa_{3}$ is the green image and financial conditions; and ④ $\kappa_{4}$ is the environmental competencies. The transportation cost ($\kappa_{2}$) is the cost attribute, and the others are beneficial attributes. The five possible suppliers of medical instrument industries $N_{i}\left(i=1,2,3,4,5\right)$ are evaluated through using P2TLNs with the four criteria by three experts $\Theta^{k}$ (expert’s weight $\gamma_{t}=\left(0.31,0.45,0.24\right)$, attributes weight $\vartheta_{j}={\left(0.2,0.31,0.23,0.26\right)}^{T}$), which are listed in Table 1, Table 2 and Table 3.

The following steps are used to select the optimal supplier of medical instrument industries by using the designed P2TL-Taxonomy method:

**Step 1:** Shift cost attribute $\kappa_{2}$ into beneficial attribute. If the cost attribute value is $\langle \left(s_{\sigma },\psi \right),\left(u_{o},v_{o}\right)\rangle$, then the corresponding beneficial attribute value is $\langle \left(s_{\sigma },\psi \right),\left(v_{o},u_{o}\right)\rangle$ (See Table 4, Table 5 and Table 6).

**Step 2:** Construct the evaluation matrix $X^{\left(3\right)}={\left(x_{ij}^{3}\right)}_{5\times 4}\left(i=1,2,3,4,5,j=1,2,3,4\right)$ of each DM as in Table 4, Table 5 and Table 6. Based on Table 4, Table 5 and Table 6 and Equation (9), the group P2TLN decision matrix is computed and presented in Table 7.

**Step 3.** Equations (11) and (12) are used to calculate the mean ${\bar{x}}_{j}$ and standard deviation $S_{j}$ of the attributes.

$$\begin{array}{l} {\bar{x}}_{1}=\langle \left(S_{3},0.098\right),(0.58,0.5964)\rangle ,\;{\bar{x}}_{2}=\langle \left(S_{3},-0.118\right),(0.5808,0.433)\rangle ,\;\\ {\bar{x}}_{3}=\langle \left(S_{4},-0.364\right),(0.6085,0.5464)\rangle ,{\bar{x}}_{4}=\langle \left(S_{3},0.334\right),(0.4998,0.6003)\rangle \end{array}$$

$$S_{1}=0.0835\;,\;S_{2}=0.1117\;,\;S_{3}=0.1455\;,\;S_{4}=0.0821\;$$

**Step 4:** The standard matrix $A_{ij}$:

$$A_{ij}={\left[a_{ij}\right]}_{5\times 4}=\left[\begin{array}{cccc} 1.0835 & 0.4014 & 0.3772 & 0.3509\\ 1.6686 & 1.6808 & 0.3136 & 1.1296\\ 0.6862 & 0.3935 & 1.4131 & 0.4300\\ 0.7157 & 1.3447 & 1.5444 & 1.4746\\ 0.2422 & 0.2253 & 0.6144 & 1.1142 \end{array}\right]$$

**Step 5:** The composite distances matrix $C_{ab}$:

$$C_{ab}={\left[c_{ab}\right]}_{5\times 5}=\left[\begin{array}{ccccc} - & 0.8571 & 0.5292 & 0.9718 & 0.5617\\ 0.8571 & - & 1.0546 & 0.7720 & 1.0546\\ 0.5292 & 1.0546 & - & 0.7539 & 0.5626\\ 0.9718 & 0.7720 & 0.7539 & - & 0.8160\\ 0.5617 & 1.0546 & 0.5626 & 0.8160 & - \end{array}\right]$$

**Step 6:** Homogenizing the alternatives:

$$Q_{1}=0.5292,\;Q_{2}=0.7720,\;Q_{3}=0.5292,\;Q_{4}=0.7539,\;Q_{5}=0.5617\;Q=\bar{Q}\pm 2S_{Q}=0.6292\pm 2\times (0.1100)=0.6292\pm 0.22$$

Therefore, all values of the composite distance matrix are in this range, and the alternatives are homogeneous.

**Step 7:** The development pattern:

In view of $\kappa_{2}$ as a cost attribute, $\kappa_{1},$ $\kappa_{2},$ $\kappa_{3}$ and $\kappa_{4}$ are benefit attributes, so the ideal values of the alternatives based on the standard matrix $A_{ij}$ are as follows:

$$z_{o1}=0.2422,\;z_{o2}=1.6808,\;z_{o3}=1.5444,\;z_{o4}=1.4746$$

$$C_{1o}=1.1360,\;C_{2o}=0.8867,\;C_{3o}=0.9170,\;C_{4o}=0.2826,\;C_{5o}=0.9431$$

**Step 8:** The final ranking of alternatives:

$$C_{o}={\bar{C}}_{io}+2S_{C_{io}}=1.7461$$

$$F_{1}=0.6506,\;F_{2}=0.5078,\;F_{3}=0.5251,\;F_{4}=0.1618,\;F_{5}=0.5401$$

Finally, the optimal supplier of medical instrument industries is $N_{4}$ and the alternatives are ranked as follows:

$$N_{4}>N_{2}>N_{3}>N_{5}>N_{1}$$

### 4.2. Comparative Analyses 

**Figure 1:**
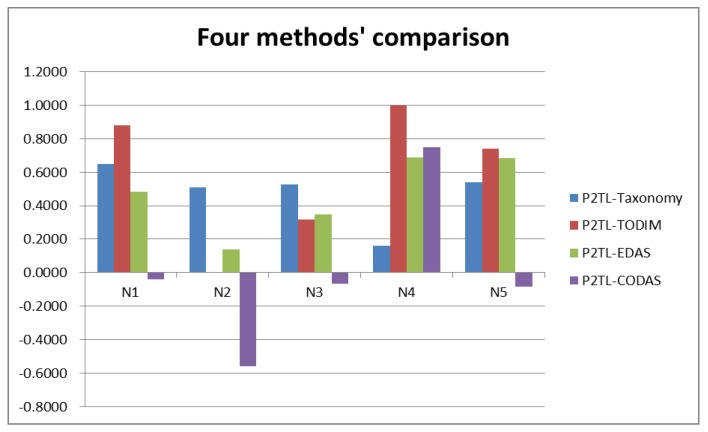
Comparison of the four methods.

A comparative analysis is also performed in this section to demonstrate the stability of the ranking result. We will compare our proposed P2TL-Taxonomy model with the P2TLWA and P2TLWG operators defined by Wei [23], the P2TL-TODIM method [23], the P2TL-EDAS (Pythagorean 2-tuple linguistic-Evaluation based on Distance from Average Solution) method and the P2TL-CODAS method. The comparison results of different methods are listed in Table 8 and Figure 1.

It is clear from Table 8 and Figure 1 that the results are slightly different in ranking of alternatives but the best alternative is always $N_{4}$ by comparing the values of our proposed P2TL-Taxonomy method with the P2TLWA/P2TLWG operators, P2TL-TODIM method, P2TL-EDAS method and P2TL-CODAS method. Notably, in practical MADM problems, the P2TL-Taxonomy method is very suitable for grading and comparing the advantages and practicalities of different alternatives according to the attributes of the research.

All of these methods have their advantages: (1) P2TLWA operators emphasise the group influences; (2) P2TLWG operators emphasise individual influences; (3) the P2TL-TODIM method based on the prospect theory is a kind of method that considers the influence of the experts’ psychological behavior factors on the decision results; (4) the P2TL-EDAS method uses the P2TL positive and negative distances from the average solution rather than the distances from the ideal solutions to any alternatives as are used in other methods; (5) the P2TL-CODAS method has the precious characteristics of taking the conflicting attributes into consideration and could be more accurate and effective in MAGDM issues; (6) our proposed P2TL-Taxonomy method is very suitable for grading and comparing the advantages and practicalities of different alternatives according to the attributes of the research.

## 5. Conclusions

The P2TLSs can reflect uncertain or fuzzy information well and solve these kind of problems, and the original Taxonomy is very appropriate for comparing different alternatives with respect to their advantages from studied attributes. In this paper, a Taxonomy method is designed for MAGDM with P2TLNs. First, the basic definition of P2TLNs is introduced. Second, the optimal alternative(s) are determined by calculating the smallest development attribute values with P2TLNs from the Pythagorean 2-tuple linguistic positive ideal solution (P2TLPIS). Finally, a numerical example for supplier selection in medical instrument industries is used to illustrate the use of the proposed method. This comparative study shows that the proposed MAGDM algorithm is feasible. This method is very effective and useful for decision making issues.

The main contributions of this study are three fold: (1) the Pythagorean 2-tuple linguistic Taxonomy (P2TL-Taxonomy) method is designed to tackle the Pythagorean 2-tuple linguistic MAGDM issues; (2) a case study for supplier selection in medical instrument industries is designed to show the developed approach; and (3) some comparative studies are provided with the existing methods to give effect to the rationality of P2TL-Taxonomy. Finally, the proposed method can also contribute to the successful selection of suitable alternatives in other selection issues.

In the future, the proposed method can be expanded to deal with other decision-making issues [39,40,41,42,43], such as the selection of green suppliers [44,45,46,47,48,49,50], the location of waste disposal stations, and so on, and the developed approaches can also be extended to further unpredictable and uncertain information. Further studies could also aim at applying different distance measures in the decision making issues and the Monte Carlo simulations could also be carried out in order to identify the best performing settings.

## Figures and Tables

**Table 1:** The Pythagorean 2-tuple linguistic number (P2TLN) decision matrix by the first expert $\Theta^{\left(1\right)}$.

Alternatives	$\kappa_{1}$	$\kappa_{2}$	$\kappa_{3}$	$\kappa_{4}$
$N_{1}$	<(s_3_, 0), (0.6, 0.5)>	<(s_6_, 0), (0.3, 0.3)>	<(s_1_, 0), (0.6, 0.6)>	<(s_4_, 0), (0.2, 0.3)>
$N_{2}$	<(s_1,_ 0), (0.3, 0.8)>	<(s_2_, 0), (0.6, 0.4)>	<(s_4_, 0), (0.6, 0.3)>	<(s_0_, 0), (0.1, 0.6)>
$N_{3}$	<(s_3_, 0), (0.4, 0.6)>	<(s_1_, 0), (0.3, 0.4)>	<(s_5_, 0), (0.6, 0.3)>	<(s_3_, 0), (0.7, 0.4)>
$N_{4}$	<(s_4_, 0), (0.6, 0.7)>	<(s_6_, 0), (0.4, 0.2)>	<(s_6_, 0), (0.8, 0.4)>	<(s_1_, 0), (0.4, 0.6)>
$N_{5}$	<(s_2_, 0), (0.5, 0.5)>	<(s_4_, 0), (0.7, 0.4)>	<(s_5_, 0), (0.6, 0.5)>	<(s_6_, 0), (0.4, 0.5)>

**Table 2:** The P2TLN decision matrix by the second expert $\Theta^{\left(2\right)}$.

Alternatives	$\kappa_{1}$	$\kappa_{2}$	$\kappa_{3}$	$\kappa_{4}$
$N_{1}$	<(s_6_, 0), (0.8, 0.7)>	<(s_1_, 0), (0.5, 0.8)>	<(s_6_, 0), (0.3, 0.3)>	<(s_5_, 0), (0.1, 0.7)>
$N_{2}$	<(s_1_, 0), (0.6, 0.5)>	<(s_0_, 0), (0.2, 0.2)>	<(s_3_, 0), (0.8, 0.6)>	<(s_3_, 0), (0.5, 0.3)>
$N_{3}$	<(s_3_, 0), (0.4, 0.2)>	<(s_3_, 0), (0.1, 0.8)>	<(s_1_, 0), (0.3, 0.6)>	<(s_5_, 0), (0.1, 0.7)>
$N_{4}$	<(s_4_, 0), (0.7, 0.7)>	<(s_5_, 0), (0.2, 0.6)>	<(s_4_, 0), (0.7, 0.1)>	<(s_2_, 0), (0.8, 0.5)>
$N_{5}$	<(s_4_, 0), (0.8, 0.3)>	<(s_3_, 0), (0.3, 0.6)>	<(s_5_, 0), (0.5, 0.6)>	<(s_6_, 0), (0.4, 0.7)>

**Table 3:** The P2TLN decision matrix by the third expert $\Theta^{\left(3\right)}$.

Alternatives	$\kappa_{1}$	$\kappa_{2}$	$\kappa_{3}$	$\kappa_{4}$
$N_{1}$	<(s_1_, 0), (0.4, 0.3)>	<(s_0_, 0), (0.2, 0.6)>	<(s_5_, 0), (0.8, 0.6)>	<(s_3_, 0), (0.2, 0.1)>
$N_{2}$	<(s_4_, 0), (0.2, 0.6)>	<(s_2_, 0), (0.5, 0.5)>	<(s_3_, 0), (0.6, 0.6)>	<(s_2_, 0), (0.7, 0.5)>
$N_{3}$	<(s_1_, 0), (0.3, 0.8)>	<(s_5_, 0), (0.6, 0.5)>	<(s_0_, 0), (0.1, 0.5)>	<(s_4_, 0), (0.4, 0.7)>
$N_{4}$	<(s_6_, 0), (0.1, 0.5)>	<(s_2_, 0), (0.7, 0.7)>	<(s_4_, 0), (0.5, 0.3)>	<(s_1_, 0), (0.6, 0.6)>
$N_{5}$	<(s_2_, 0), (0.3, 0.8)>	<(s_4_, 0), (0.7, 0.6)>	<(s_1_, 0), (0.4, 0.8)>	<(s_2_, 0), (0.6, 0.3)>

**Table 4:** The P2TLN normalized decision matrix by the first expert $\Theta^{\left(1\right)}$.

Alternatives	$\kappa_{1}$	$\kappa_{2}$	$\kappa_{3}$	$\kappa_{4}$
$N_{1}$	<(s_3_, 0), (0.6, 0.5)>	<(s_6_, 0), (0.3, 0.3)>	<(s_1_, 0), (0.6, 0.6)>	<(s_4_, 0), (0.2, 0.3)>
$N_{2}$	<(s_1,_ 0), (0.3, 0.8)>	<(s_2_, 0), (0.4, 0.6)>	<(s_4_, 0), (0.6, 0.3)>	<(s_0_, 0), (0.1, 0.6)>
$N_{3}$	<(s_3_, 0), (0.4, 0.6)>	<(s_1_, 0), (0.4, 0.3)>	<(s_5_, 0), (0.6, 0.3)>	<(s_3_, 0), (0.7, 0.4)>
$N_{4}$	<(s_4_, 0), (0.6, 0.7)>	<(s_6_, 0), (0.2, 0.4)>	<(s_6_, 0), (0.8, 0.4)>	<(s_1_, 0), (0.4, 0.6)>
$N_{5}$	<(s_2_, 0), (0.5, 0.5)>	<(s_4_, 0), (0.4, 0.7)>	<(s_5_, 0), (0.6, 0.5)>	<(s_6_, 0), (0.4, 0.5)>

**Table 5:** The P2TLN normalized decision matrix by the second expert $\Theta^{\left(2\right)}$.

Alternatives	$\kappa_{1}$	$\kappa_{2}$	$\kappa_{3}$	$\kappa_{4}$
$N_{1}$	<(s_6_, 0), (0.8, 0.7)>	<(s_1_, 0), (0.8, 0.5)>	<(s_6_, 0), (0.3, 0.3)>	<(s_5_, 0), (0.1, 0.7)>
$N_{2}$	<(s_1_, 0), (0.6, 0.5)>	<(s_0_, 0), (0.2, 0.2)>	<(s_3_, 0), (0.8, 0.6)>	<(s_3_, 0), (0.5, 0.3)>
$N_{3}$	<(s_3_, 0), (0.4, 0.2)>	<(s_3_, 0), (0.8, 0.1)>	<(s_1_, 0), (0.3, 0.6)>	<(s_5_, 0), (0.1, 0.7)>
$N_{4}$	<(s_4_, 0), (0.7, 0.7)>	<(s_5_, 0), (0.6, 0.2)>	<(s_4_, 0), (0.7, 0.1)>	<(s_2_, 0), (0.8, 0.5)>
$N_{5}$	<(s_4_, 0), (0.8, 0.3)>	<(s_3_, 0), (0.6, 0.3)>	<(s_5_, 0), (0.5, 0.6)>	<(s_6_, 0), (0.4, 0.7)>

**Table 6:** The P2TLN normalized decision matrix by the third expert $\Theta^{\left(3\right)}$.

Alternatives	$\kappa_{1}$	$\kappa_{2}$	$\kappa_{3}$	$\kappa_{4}$
$N_{1}$	<(s_1_, 0), (0.4, 0.3)>	<(s_0_, 0), (0.6, 0.2)>	<(s_5_, 0), (0.8, 0.6)>	<(s_3_, 0), (0.2, 0.1)>
$N_{2}$	<(s_4_, 0), (0.2, 0.6)>	<(s_2_, 0), (0.5, 0.5)>	<(s_3_, 0), (0.6, 0.6)>	<(s_2_, 0), (0.7, 0.5)>
$N_{3}$	<(s_1_, 0), (0.3, 0.8)>	<(s_5_, 0), (0.5, 0.6)>	<(s_0_, 0), (0.1, 0.5)>	<(s_4_, 0), (0.4, 0.7)>
$N_{4}$	<(s_6_, 0), (0.1, 0.5)>	<(s_2_, 0), (0.7, 0.7)>	<(s_4_, 0), (0.5, 0.3)>	<(s_1_, 0), (0.6, 0.6)>
$N_{5}$	<(s_2_, 0), (0.3, 0.8)>	<(s_4_, 0), (0.6, 0.7)>	<(s_1_, 0), (0.4, 0.8)>	<(s_2_, 0), (0.6, 0.3)>

**Table 7:** The group Pythagorean 2-tuple linguistic decision matrix $X_{ij}$.

Alternatives	$\kappa_{1}$	$\kappa_{2}$
$N_{1}$	<(s_4_, −0.13), (0.6875,0.638)>	<(s_2_, 0.31), (0.6701,0.4975)>
$N_{2}$	<(s_2_, −0.28), (0.4618,0.6476)>	<(s_1_, 0.1), (0.3632,0.4104)>
$N_{3}$	<(s_3_, −0.48),(0.3791,0.4594)>	<(s_3_, −0.14), (0.6646,0.3139)>
$N_{4}$	<(s_4_, 0.48),(0.5987,0.7212)>	<(s_5_, −0.41), (0.5593,0.4449)>
$N_{5}$	<(s_4_, −0.13), (0.6875,0.638)>	<(s_2_, 0.31), (0.6701,0.4975)>
**Alternatives**	$\kappa_{3}$	$\kappa_{4}$
$N_{1}$	<(s_4_, 0.21), (0.589, 0.5146)>	<(s_4_, 0.21), (0.1631, 0.4901)>
$N_{2}$	<(s_3_, 0.31), (0.7113, 0.7029)>	<(s_2_, −0.17), (0.5048, 0.4926)>
$N_{3}$	<(s_2_, 0), (0.4091, 0.6729)>	<(s_4_, 0.14), (0.4745, 0.7818)>
$N_{4}$	<(s_5_, −0.38), (0.7056, 0.2658)>	<(s_1_, 0.45), (0.6801, 0.6476)>
$N_{5}$	<(s_4_, 0.21), (0.589, 0.5146)>	<(s_4_, 0.21), (0.1631, 0.4901)>

**Table 8:** Rank of Alternatives.

Methods	Order
P2TLWA	$N_{4}>N_{1}>N_{5}>N_{3}>N_{2}$
P2TLWG	$N_{4}>N_{5}>N_{1}>N_{3}>N_{2}$
P2TL-TODIM	$N_{4}>N_{1}>N_{5}>N_{3}>N_{2}$
P2TL-EDAS	$N_{4}>N_{5}>N_{1}>N_{3}>N_{2}$
P2TL-CODAS	$N_{4}>N_{1}>N_{3}>N_{5}>N_{2}$
P2TL-Taxonomy	$N_{4}>N_{2}>N_{3}>N_{5}>N_{1}$

P2TLWA: Pythagorean 2-tuple linguistic weighted average; P2TLWG: Pythagorean 2-tuple linguistic weighted geometric; P2TL-TODIM: Pythagorean 2-tuple linguistic TODIM; P2TL-EDAS: Pythagorean 2-tuple linguistic-Evaluation based on Distance from Average Solution; P2TL-CODAS: Pythagorean 2-tuple linguistic-COmbinative Distance-based Assessment.
